# Performance of ChatGPT in the In-Training Examination for Anesthesiology and Pain Medicine Residents in South Korea: Observational Study

**DOI:** 10.2196/56859

**Published:** 2024-09-16

**Authors:** Soo-Hyuk Yoon, Seok Kyeong Oh, Byung Gun Lim, Ho-Jin Lee

**Affiliations:** 1 Department of Anesthesiology and Pain Medicine Seoul National University Hospital Seoul National University College of Medicine Seoul Republic of Korea; 2 Department of Anesthesiology and Pain Medicine Korea University Guro Hospital Korea University College of Medicine Seoul Republic of Korea

**Keywords:** AI tools, problem solving, anesthesiology, artificial intelligence, pain medicine, ChatGPT, health care, medical education, South Korea

## Abstract

**Background:**

ChatGPT has been tested in health care, including the US Medical Licensing Examination and specialty exams, showing near-passing results. Its performance in the field of anesthesiology has been assessed using English board examination questions; however, its effectiveness in Korea remains unexplored.

**Objective:**

This study investigated the problem-solving performance of ChatGPT in the fields of anesthesiology and pain medicine in the Korean language context, highlighted advancements in artificial intelligence (AI), and explored its potential applications in medical education.

**Methods:**

We investigated the performance (number of correct answers/number of questions) of GPT-4, GPT-3.5, and CLOVA X in the fields of anesthesiology and pain medicine, using in-training examinations that have been administered to Korean anesthesiology residents over the past 5 years, with an annual composition of 100 questions. Questions containing images, diagrams, or photographs were excluded from the analysis. Furthermore, to assess the performance differences of the GPT across different languages, we conducted a comparative analysis of the GPT-4’s problem-solving proficiency using both the original Korean texts and their English translations.

**Results:**

A total of 398 questions were analyzed. GPT-4 (67.8%) demonstrated a significantly better overall performance than GPT-3.5 (37.2%) and CLOVA-X (36.7%). However, GPT-3.5 and CLOVA X did not show significant differences in their overall performance. Additionally, the GPT-4 showed superior performance on questions translated into English, indicating a language processing discrepancy (English: 75.4% vs Korean: 67.8%; difference 7.5%; 95% CI 3.1%-11.9%; *P*=.001).

**Conclusions:**

This study underscores the potential of AI tools, such as ChatGPT, in medical education and practice but emphasizes the need for cautious application and further refinement, especially in non-English medical contexts. The findings suggest that although AI advancements are promising, they require careful evaluation and development to ensure acceptable performance across diverse linguistic and professional settings.

## Introduction

ChatGPT is an artificial intelligence (AI) service for conversations based on the generated pretrained transformer and a large-scale generative language model [1]. Since the release of ChatGPT, numerous attempts have been made to apply it in health care practices [2]. In this context, its medical knowledge and thinking skills have been evaluated through a range of medical examinations including the US Medical Licensing Examination and various specialty examinations. The results indicate a performance close to the passing threshold [3]. In the field of anesthesiology, ChatGPT has been evaluated using questions from several question banks designed for English-language board examination preparation. However, doubts remain regarding their ability to complete board examinations [4,5].

GPT-4 is the successor of GPT-3.5, which formed the basis of ChatGPT after its launch. OpenAI, the developer of ChatGPT, reported that GPT-4 not only outperformed GPT-3.5 but also often scored higher than most human test-takers, demonstrating a particularly strong performance in languages other than English [6]. Indeed, in previous studies using written board examinations for neurosurgery and ophthalmology, GPT-4 exhibited a significantly higher proportion of correct responses compared to GPT-3.5 [7,8]. The superiority of GPT-4 over GPT-3.5 was also noted in the field of anesthesiology, as assessed using 27 questions from the Royal College of Anaesthetists [9]. Furthermore, a comparative study evaluating the performance of GPT-3.5 and GPT-4 on the Japanese Medical Licensing Examination revealed that GPT-4 achieved a significantly higher rate of correct responses [10], indicating its advanced performance in non-English languages.

Given the emergence and development of ChatGPT, it is crucial to examine the knowledge levels and reasoning abilities of AI language models in the fields of anesthesiology and pain medicine in Korea to estimate their potential to aid medical professionals. However, to date, no study has explored the performance of ChatGPT in the fields of anesthesiology and pain medicine in a Korean language context. Therefore, this study aimed to investigate the performance of ChatGPT, including both GPT-3.5 and GPT-4, using the in-training examination administered by the Korean Society of Anesthesiologists (KSA). This study also aimed to compare the performance of ChatGPT with that of CLOVA X, a new generative AI service in South Korea.

## Methods

### Data Source and Contents

This study evaluated the performance of AI services by using the metric “number of correct answers/number of questions” [3], using the KSA in-training examinations from 2018 to 2022, each comprising 100 annual questions. The KSA conducts annual in-training tests for residents to assess their readiness and prepare them for specialist certification examinations. Beginning in 2019, a cutoff point system was introduced: if an examinee scored below a certain threshold for each year’s grade, they were considered to have failed and were required to retake the examination. The cutoff points are set at 50 in the first year of training, 55 in the second year, and 60 in the third and fourth years. The full texts of the questionnaires, correct answers, and commentaries provided by the society are accessible only to members via official websites [11]. Each question consisted of one query and five choices, each with one correct answer. Because ChatGPT only accepts text as input, we excluded questions with images, diagrams, or photographs within the question content.

To compare the performance of ChatGPT with that of the actual examinees, we requested anonymized data from the Training and Education Committee of the KSA on the scores achieved by residents over the past 5 years, both overall and for each training year.

Initially, we focused solely on evaluating the performance of ChatGPT. However, a new generative AI service, CLOVA X, was launched in South Korea by Naver Corporation in August 2023 during our study period; therefore, we decided to expand our investigation to include an examination of its performance. CLOVA X was developed based on the Korean large language model HyperCLOVA X. HyperCLOVA X was trained on a vast corpus of high-quality data primarily sourced from Korean text content. This makes the training data particularly rich in terms of Korean culture and lifestyle, unlike the more diverse multilingual data sets used for ChatGPT. In addition, HyperCLOVA X uses specific alignment techniques, such as supervised fine-tuning and reinforcement learning from human feedback, to enhance its ability to follow instructions and align with human values [12].

### Ethical Considerations

The ethical review of the study was exempted by the institutional review board of Seoul National University Hospital (E-2308-102-1459). This study used only in-training examinations that are already available on the KSA website and did not involve human participants or use any personal information.

### Testing Process

We compared the performances of GPT-3.5, GPT-4, and CLOVA X in solving problems in the fields of anesthesiology and pain medicine using the following process: to ensure that both models were tested under identical conditions, the following command in Korean was entered before posing the questions: “(Translated) Below are the in-training assessment questions for the specialty of anesthesiology and pain medicine. Please complete the questions and describe your solution in detail. There is only one answer for each question” (Figure S1A in Multimedia Appendix 1). Previously, there were instances in which multiple answers were provided by ChatGPT when the prompt did not explicitly state a single correct answer. In addition, answers were sometimes provided without explanation, when the prompt did not request detailed steps. Therefore, we implemented these commands to address these issues. The included questions were then individually entered into the prompt in the order of their question numbers, exactly as they were written in Korean (Multimedia Appendix 1). This is because we determined that within the same window, previously entered questions could influence the answers to subsequent questions. If a question or choice included a table, we transcribed the content and maintained the same arrangement, using spaces and hyphens. After completing the set of questions, a new window was opened, the same command was entered, and questions from another year were entered. This process was identical for both ChatGPT and CLOVA X.

For each question input, we recorded the answers chosen by GPT-3.5, GPT-4, and CLOVA X and the explanation for the selection. After completing the problem-solving process for all the questions in both models, we compared their responses to the answer keys provided by the KSA. An answer was recorded as correct if the first response matched the actual response. It was recorded as incorrect if no answer was selected, if the answer was incorrect, or if multiple answers were selected, even if the correct answer was among them. After scoring, we calculated the overall and yearly scores for GPT-3.5, GPT-4, and CLOVA X, as well as the percentage of questions answered correctly relative to the total number of questions.

To compare the performance of GPT-4 in Korean and English, we translated the questions into English and conducted additional problem-solving. This process was conducted in 2 stages. First, the English translation was initially performed by inputting the original Korean questions one by one, along with the command, “Please translate the following into English.” All questions included in this study were translated, and the translated texts were recorded separately (Figure S1A in Multimedia Appendix 2). Two authors (SHY and HJL) reviewed the accuracy of English translations. In the second stage, we entered the initial instruction commands used for the Korean questions in English into a new window (Figure S1B in Multimedia Appendix 2), followed by the translated English questions individually (Figure S1C in Multimedia Appendix 2). The process of answering the questions, recording the answers and explanations, and scoring was identical to that used for the Korean questions.

Two authors (SHY and HJL) conducted the task of having AI services solve problems, and all authors reviewed the results. Two authors (SHY and HJL) were using the paid version of ChatGPT-4 at their own expense, independently of this study. ChatGPT-3.5 and CLOVA X were used free of charge. Therefore, no additional costs were incurred when using the three programs.

### Outcome Measure and Analysis

The primary outcome of this study was to assess the performance difference between GPT-3.5, GPT-4, and CLOVA-X, as measured by the overall score on the 5 years of in-training examination for residents of anesthesiology and pain medicine in Korea. Secondary outcomes included performance on the ChatGPT and CLOVA-X according to the examination year, subfields within anesthesiology and pain medicine, inclusion of clinical cases, and level of logical thinking required by the questions. Additionally, the performance of GPT-4 on the English-translated questions was compared to its performance on the original Korean version.

The analytical methods used in this study were first used to compare the overall performances of GPT-3.5, GPT-4, and CLOVA X for each year. As a performance reference, we calculated the mean and SD of the examinees’ scores both overall and for each training year. However, a direct comparison of the scores was not possible because the study excluded questions involving images, diagrams, and photographs. Second, the questions were categorized into the subfields of anesthesiology and pain medicine following the taxonomy outlined by the KSA. Third, we classified the questions based on the inclusion of clinical cases or the level of logical thinking required (Figure S1B-D in Multimedia Appendix 1). A question was classified as containing a clinical case if it described a specific situation involving patient information, such as demographics, medical history, surgery, and anesthesia, requiring the use of this information to answer the question. If the question dealt only with theoretical knowledge or if there was some mention of a patient but it was not necessary to apply this information to answer the question, we classified the question as not containing a clinical case. The level of logical thinking was categorized as either first-order or higher-order problem-solving based on a previous study design that evaluated the performance of GPT-3.5 and GPT-4 on the self-assessment examination of neurosurgery [7]. A question was classified as first order if it required direct use of the conditions or circumstances of the question, simple recall of facts, selection of an answer from a set of choices, or determination of the truth or falsity of each option. When a question required additional logical steps to select the correct answer, such as estimating a diagnosis, applying guidelines, or calculating with formulas, it was classified in the higher order. Fourth, we compared the differences in GPT-4 performance between the original Korean questions and their English-translated versions. Additionally, we measured self-agreement, which refers to the number and percentage of questions for which ChatGPT chose the same answer in Korean and English, irrespective of the accuracy of the response.

During the revision process, we further analyzed the explanations for the incorrect answers of each model. A classification system from a previous study was used to categorize the reasons for each incorrect choice as logical, informational, or statistical errors [13]. In cases where two errors occurred simultaneously, both errors were identified. This process was conducted independently by two authors (SHY and HJL), and discrepancies in labeling were resolved through a post hoc discussion involving all authors.

### Statistics

When comparing the performances of GPT-3.5, GPT-4, and CLOVA X in Korean, we used the Cochran *Q* test; in cases where there was a significant difference among the 3 tools, the comparison between the two groups was investigated by calculating the minimum required difference for a significant difference between the 2 groups [14]. The significance level of Cochran *Q* test for the three language models was 0.05, while a Bonferroni correction was applied to set the significance level to 0.017 when comparing the two groups, considering that there were three combinations for comparisons. Additionally, although we used the KSA classification to compare the performances of GPT-3.5, GPT-4, and CLOVA-X across various subfields, we recognized that the number of questions per field was too limited for a statistical comparison. Descriptive statistics were used to analyze these factors. A chi-square test was conducted to compare the inclusion of clinical cases and the level of logical thinking in the questions. Finally, to compare the performance of GPT-4 in Korean and English, we used McNemar’s test and calculated Poisson 95% CIs for the two performances. All statistical analyses were performed using MedCalc Statistical Software (version 18.6; MedCalc Software bvba).

## Results

A total of 398 questions were included in the analysis, selected from a set of 500 questions used over the past 5 years, excluding those containing images, diagrams, or photographs. The performances of GPT-3.5, GPT-4, and CLOVA X are presented in Table 1. The overall performance of GPT-4 (67.8%) was significantly higher than that of GPT-3.5 (37.7%) and CLOVA X (37.2%), surpassing the minimum required difference of 9.1% in Cochran *Q* test. However, GPT-3.5 and CLOVA X did not show significant differences in their overall performance. In the year-by-year analysis, GPT-4 consistently showed a significantly higher performance than GPT-3.5 and CLOVA-X, except in 2022 when only the difference between GPT-4 and GPT-3.5 was significant. Multimedia Appendix 3 shows the actual scores of Korean anesthesiology residents in 2022, 2021, and 2019. However, due to the unavailability of data for 2018 and the inapplicability of the 2020 data for estimating the mean and SD of the residents’ scores, these years were excluded from the analysis.

**Table 1:** Performances of the models in overall and yearly examinations.

Year (questions)^a^	GPT-3.5^b^, n (%)	GPT-4^b^, n (%)	CLOVA X^b^, n (%)	*P* value^c^	GPT-4 versus GPT-3.5^c^	GPT-4 versus CLOVA X^c^	GPT-3.5 versus CLOVA X^c^
2022 (n=72)	20 (28)	49 (68)	34 (47)	<.001	S^d^	N/S^e^	N/S
2021 (n=74)	29 (39)	51 (69)	23 (31)	<.001	S	S	N/S
2020 (n=79)	28 (35)	54 (68)	22 (28)	<.001	S	S	N/S
2019 (n=85)	36 (42)	53 (62)	33 (39)	.001	S	S	N/S
2018 (n=88)	37 (42)	63 (72)	36 (41)	<.001	S	S	N/S
Total (n=398)	150 (37.7)	270 (67.8)	148 (37.2)	<.001	S	S	N/S

^a^Number of questions included in the overall and yearly examinations is presented in parentheses.

^b^Performances of ChatGPT and CLOVA X are presented as the number of correct answers for each examination, along with the percentage of correct answers out of the total number of questions in parentheses.

^c^Cochran *Q* test was conducted to compare the performance of GPT-3.5, GPT-4, and CLOVA X, and the *P* values are presented. In multiple comparisons of the two models, significance determined at a *P* value of .017 using Bonferroni correction was denoted as S or N/S.

^d^S: significant.

^e^N/S: not significant.

Table 2 presents a comparison of the performances of GPT-3.5, GPT-4, and CLOVA-X in each specific subfield of anesthesiology. A total of 21 subfields were examined based on the taxonomy of the KSA. The highest-scoring subfield was geriatric anesthesia in GPT-3.5 (58.8%), GPT-4 (88.2%), and CLOVA X (64.7%). The lowest scoring subfield was “neuromuscular blocking agents” for GPT-3.5 and CLOVA X (17.6%), and “anesthesia equipment and monitoring” for GPT-4 (37.5%).

**Table 2:** Performance for each subfield in anesthesiology and pain medicine.

Subfields (questions)^a^	GPT-3.5^b^, n (%)	GPT-4^b^, n (%)	CLOVA X^b^, n (%)
Medical ethics (n=5)	1 (20)	3 (60)	2 (40)
Preanesthetic care (n=11)	5 (46)	5 (46)	5 (46)
Anesthesia equipment and monitoring (n=16)	4 (25)	6 (38)	8 (50)
Transplant anesthesia (n=19)	5 (26)	13 (68)	4 (21)
Inhalation anesthesia (n=21)	5 (24)	14 (67)	9 (43)
Obstetric anesthesia (n=25)	11 (44)	15 (60)	9 (36)
Pediatric anesthesia (n=24)	8 (33)	17 (71)	9 (38)
Ambulatory anesthesia (n=11)	6 (55)	8 (73)	3 (27)
Neuromuscular blocking agents (n=17)	3 (18)	12 (71)	3 (18)
Geriatric anesthesia (n=17)	10 (59)	15 (88)	11 (65)
Regional anesthesia (n=22)	11 (50)	15 (68)	7 (32)
Neuro-anesthesia (n=20)	9 (45)	16 (80)	11 (55)
Anesthetic pharmacology (n=11)	3 (27)	7 (64)	3 (27)
Intravenous anesthesia (n=13)	5 (39)	5 (39)	6 (46)
Cardiac anesthesia (n=14)	3 (21)	10 (71)	3 (21)
Thoracic anesthesia (n=17)	5 (29)	9 (53)	4 (24)
Fluids and transfusion (n=19)	6 (32)	14 (74)	6 (32)
Cardio-pulmonary resuscitation (n=17)	7 (41)	13 (77)	5 (29)
Pain clinic (n=57)	20 (35)	42 (74)	24 (42)
Intensive care unit (n=31)	17 (55)	23 (74)	12 (39)
Sedation or anesthesia outside the operating theater (n=11)	6 (55)	8 (73)	4 (36)
Total (n=398)	150 (37.7)	270 (67.8)	148 (37.2)

^a^Number of questions in each subfield is presented in parentheses.

^b^Performances of ChatGPT and CLOVA X are presented as the number of correct answers for each subfield along with the percentage of correct answers out of the total number of questions in parentheses.

Table 3 presents a comparison of the performances of GPT-3.5, GPT-4, and CLOVA X based on the question type. The models exhibited no significant performance differences when clinical cases were included. However, in terms of the level of logical thinking, GPT-3.5 and CLOVA X showed no significant difference, whereas GPT-4 showed a significantly higher performance for higher-order questions than for first-order questions (77% vs 64.2%; *P*=.02).

**Table 3:** Performance based on the inclusion of a clinical case and the level of logical thinking in the question.

Category and number of questions			GPT-3.5^a^, n (%)		*P* value^b^		GPT-4^a^, n (%)		*P* value^b^		CLOVA X^a^, n (%)		*P* value^b^
**Case**					.57				.20				.11
	Included (n=185)	73 (39.5)				132 (71.4)				77 (41.6)			
	Not included (n=213)	77 (36.2)				138 (64.8)				71 (33.3)			
**Level**					.35				.02				.09
	First-order (n=285)	112 (39.3)				183 (64.2)				98 (34.4)			
	Higher-order (n=113)	38 (33.6)				87 (77)				50 (44.2)			

^a^Performances of ChatGPT and CLOVA X are presented as the number of correct answers for each category, along with the percentage of correct answers out of the total number of questions in parentheses.

^b^A chi-square test was conducted to compare each performance of GPT-3.5, GPT-4, and CLOVA X according to the inclusion of cases and the level of logical thinking, and the *P* values are presented.

Table 4 presents the differences in GPT-4 performance between the original Korean questions and their English versions. All examination questions translated from Korean to English using ChatGPT-4 were accurate and appropriate. Overall, GPT-4 performed significantly better on English-translated questions than on Korean originals (75.4% vs 67.8%; difference 7.5%; 95% CI 3.1%-11.9%; *P*=.001). When analyzed by year, the performance was consistently higher in English than in Korean, with the difference reaching statistical significance only in 2019 (75.3% vs 62.3%; *P*=.01). Furthermore, the overall self-agreement rate between the Korean and English-translated versions was 72.6%. In 14.1% of cases, correct answers were derived only from the English-translated version, and in 6.5% of cases, correct answers were derived solely from the original Korean questions.

**Table 4:** Performance of GPT-4 on Korean and English versions.

Year (questions)^a^	Korean^b^, n (%)	English^b^, n (%)	Difference (95% CI)^c^	*P* value^c^	Correct answers in each language^d^			Self-agreement^d^, n (%)
					Both language, n (%)	Korean only, n (%)	English only, n (%)	
2022 (72)	49 (68)	54 (75)	6.9% (–4.2 to 18.1)	.33	43 (60)	6 (8)	11 (15)	49 (68)
2021 (74)	51 (69)	57 (77)	8.1% (–2.3 to 18.5)	.21	46 (62)	5 (7)	11 (15)	54 (73)
2020 (79)	54 (68)	59 (75)	6.3% (–4.4 to 17.1)	.36	47 (60)	7 (9)	12 (15)	55 (70)
2019 (85)	53 (62)	64 (75)	12.9% (3.8 to 22.0)	.01	50 (59)	3 (4)	14 (17)	62 (73)
2018 (88)	63 (72)	66 (75)	3.4% (–4.6 to 11.4)	.58	58 (66)	5 (6)	8 (9)	69 (78)
Total (398)	270 (67.8)	300 (75.4)	7.5% (3.1 to 11.9)	.001	244 (61.3)	26 (6.5)	56 (14.1)	289 (72.6)

^a^Number of questions included in the overall and yearly examinations is presented in parentheses.

^b^Performance in GPT-4 is presented as the number of correct answers for each language along with the percentage of correct answers out of the total number of questions in parentheses.

^c^McNemar’s test was conducted to compare the performance of GPT-4 in Korean and English, and the differences of proportion (95% CI) with the *P* values are presented.

^d^Other variables, such as the number of correct answers in both languages, Korean only, and English only, and the self-agreement rate of ChatGPT answers when tested in Korean and English, are presented as numbers and percentages.

Table 5 presents the categorized reasons for the incorrect answers for each model. In all models, over 70% of the incorrect answers were due to informational errors, whereas less than 10% were caused by simple logical errors.

**Table 5:** Reasons for incorrect answers.

Category^a^	GPT-3.5	GPT-4 (Korean)	GPT-4 (English)	CLOVA X
Logical error, n (%)	24 (9.7)	11 (8.6)	7 (7.1)	4 (1.6)
Information error, n (%)	183 (73.8)	107 (83.6)	86 (87.8)	185 (74.0)
Statistical error, n (%)	3 (1.2)	1 (0.8)	1 (1.0)	3 (1.2)
Logical and information errors, n (%)	38 (15.3)	9 (7.0)	4 (4.1)	58 (23.2)
Overall, n	248	128	98	250

^a^Reasons for incorrect answers by ChatGPT and CLOVA X are presented as numbers with percentages of the total number of incorrect answers in parentheses.

## Discussion

### Principal Findings

This study assessed the proficiency of ChatGPT in the fields of anesthesiology and pain medicine by analyzing its performance on in-training examinations administered to Korean anesthesiology residents over the past 5 years. Our findings revealed that GPT-4 performed better in solving Korean-language problems in this field than its predecessors, GPT-3.5 and CLOVA X, which were trained using a Korean-language database. An interesting observation emerged when examination questions originally written in Korean were translated into English. In this scenario, GPT-4 exhibits higher performance levels. This suggests an enhanced capability of GPT-4 to process and respond to questions in English compared to Korean. However, it is important to note that despite this improved performance in the English-translated examinations, GPT-4 did not meet the recommended performance level for educational tools (>95%) [15].

### Comparison to the Literature

In the fields of anesthesiology and pain medicine, the ChatGPT knowledge base has been rigorously evaluated using various practical questions. A previous report involving 1321 questions from the American Board of Anesthesiology (ABA) examination preparation book revealed that GPT-3.5 attained a correct answer rate of 56.2% [4]. A recent follow-up report with the same set of questions in GPT-4 discovered a remarkable improvement, with a correct answer rate of 72.1% [16]. In a separate evaluation using 3705 questions from the Fellowship of the Royal College of Anaesthetists Primary examination, GPT-3.5 achieved a higher correct answer rate of 69.7% [5]. Furthermore, in a study that used a mock ABA examination comprising questions from the ABA website and examination preparation book, GPT-4 was the only tool among its peers, including GPT-3.5 and Google Bard, to pass all three stages of the examination [17]. However, these studies focused on English-language questions. This study differs by examining ChatGPT’s performance on non-English questions, encompassing both translated versions and the original Korean questions. Additionally, this study provides a unique perspective by presenting the scoring results of Korean anesthesiology residents, facilitating a direct comparison between human performance and ChatGPT.

Additionally, our results reaffirmed the performance disparities of ChatGPT on English and Korean questions, as observed in recently reported studies in medicine. A notable study in the field of dermatology that used the Korean dermatology specialty certificate examination found that the English-translated version of the questions yielded significantly higher performance than the original Korean version (69.0% vs 57.0%) [18]. Another study assessed the performance differentials between GPT-3.5 and GPT-4 by translating cirrhosis-related questions into multiple languages including English, Korean, Mandarin, and Spanish [19]. This study revealed that GPT-4 consistently outperformed GPT-3.5 across all languages, with the performance gap being more pronounced in the Korean and Mandarin versions than in English. Notably, even GPT-4 demonstrated lower performance in Korean than in English, which is consistent with the trends observed in this study. The GPT-4 technical report by OpenAI provides further insight, indicating that while GPT-4’s performance in Korean surpassed that of GPT-3.5 in English (77% vs 70.1%), it fell short compared to GPT-4’s performance in English (85.5% vs 77.0%) [6]. This disparity in the language-specific performance of ChatGPT can be attributed to the predominance of English-based text in the GPT training data. This is particularly significant in the medical field, where there is more English literature than Korean literature. Consequently, the process of translating Korean questions into English for answer generation, followed by retranslation into Korean, likely affected performance. This is due to potential losses or alterations in meaning inherent in the translation process [20].

### Implications of Findings

To the best of our knowledge, this is the first study to investigate the problem-solving performance of CLOVA X using medical knowledge. Although CLOVA X is a generative AI tool developed based on the Korean large-scale language model AI HyperCLOVA X, its performance in solving anesthesiology and pain medicine problems posed in Korea was inferior to that of GPT-4 and similar to that of GPT-3.5. This likely resulted from the HyperCLOVA X being trained exclusively on Korean data. The size of medical knowledge data sets likely varies by language [21], and English is presumed to contain more extensive medical knowledge data than other languages. Therefore, while CLOVA X might have advantages in processing Korean compared with ChatGPT, its limitations in specialized medical knowledge areas could be attributed to the limitations of its training data set.

In the results of the subfields of anesthesiology and pain medicine, the highest performances were observed in “geriatric anesthesia” in all three tools, whereas the subfield with the lowest performance was “neuromuscular blocking agents” in GPT-3.5 and CLOVA X, and “anesthesia equipment and monitoring” in GPT-4. This may be because the contents on neuromuscular blocking agents, anesthesia equipment, and monitoring are generally included in specialized textbooks that are less publicly accessible. However, neuromuscular blocking agents, which showed lower performance in GPT-3.5 and CLOVA X, showed higher-than-average performances in GPT-4.0. This improvement in GPT-4.0 suggests the potential for more sophisticated language-understanding models in these specialized fields. On the other hand, for questions about anesthesia equipment and monitoring or intravenous anesthesia, CLOVA X scored higher than both GPT-3.5 and GPT-4. It can be assumed that this prominent deviation from the overall score pattern reflects the differences in the data on which each language model was trained. Although this study did not have enough questions in each subfield to investigate the differences between them, further research on the performance differences of ChatGPT or CLOVA X across specific subfields is necessary for the future use of AI in anesthesiology and pain medicine.

The results of this study indicate that GPT-4 has the potential to surpass the correct answer rate of Korean anesthesiology residents for both Korean and English examination questions, thus meeting the passing criteria. Despite this achievement, GPT-4 failed to show acceptable performance as a reliable educational tool (>95%) [3,15] and also had the following limitations stemming from the fundamental operational mechanisms of large language models such as GPT [22]. Unlike human reasoning processes, these models generate responses based on probability distributions and likely word combinations rather than a genuine understanding of the learned content. Moreover, the possibility of incorrect GPT learning could not be ignored. Consequently, despite training with large data sets, the generated responses may be erroneous. Moreover, the model’s tendency to provide plausible yet erroneous explanations for incorrect answers poses a significant risk of disseminating misinformation to anesthesiology residents. Therefore, we conclude that these models are inadequate for medical education applications, owing to their inherent limitations.

### Opportunities for Future Work

Although GPT-4 demonstrated a higher level of knowledge in solving anesthesiology problems than Korean anesthesiology residents, its performance was not sufficiently reliable to be taken at face value. This performance shortfall is primarily attributed to the lack of training data in specialized fields such as medicine. Our analysis of incorrect answers also revealed that misinformation was the most common cause of error. If accurate information from professional medical texts is included in the training data of generative AI and is continuously updated, its performance in the medical field can be improved. However, the potential legal implications of using copyrighted textbooks, such as copyright infringement [23], further complicate the prospects of incorporating specialized medical texts into generative AI training in the future. Addressing these issues is essential to enhance the medical knowledge of generative AI models.

### Limitations

This study, while pioneering in its exploration of ChatGPT performance in Korean anesthesiology questions, had several limitations. First, the representativeness of the in-training examinations for Korean anesthesiology residents as a comprehensive measure of anesthesiology knowledge remains controversial. However, this examination was selected because it is the only test that is readily accessible to Korean anesthesiologists. Second, due to the inherent limitations of ChatGPT, our analysis excluded questions that incorporated images, diagrams, or photographs. Therefore, we were unable to directly compare the actual examination results of the residents with the performance of the AI services. Additionally, this limitation hinders our ability to fully evaluate the performance of AI services during anesthesiology examinations. This exclusion also potentially limits the scope of our findings as these elements are integral to many medical questions. Third, we used a selective data set that may not have fully captured the performance of AI across a broad range of medical scenarios in the field of anesthesiology. Future research should incorporate nonselective data sets to ensure a more comprehensive and generalizable evaluation of AI performance. Ultimately, owing to these limitations, we could only investigate a partial aspect of AI performance in understanding anesthesiology knowledge. Despite these limitations, this study is the first to assess the capabilities of ChatGPT in handling anesthesiology questions in Korean. We expect our findings to stimulate discussion and consideration among Korean anesthesiologists regarding the potential roles and limitations of AI tools, such as ChatGPT, in the field of anesthesiology. In addition, by demonstrating performance differences in GPT in English and Korean, this study raises the issue of narrowing the performance gap across different languages.

### Conclusions

In summary, this study demonstrated that, although GPT-4 is advanced compared to its predecessors in processing Korean anesthesiology examination questions, it has yet to reach a level of reliability that would justify its use as a standalone educational tool in the medical domain. Specifically, our research highlights the significant performance disparity between English and Korean ChatGPT outputs, drawing attention to the challenges inherent in evaluating proficiency in non-English medical content. This investigation of the capabilities of ChatGPT in Korean anesthesiology is a pioneering effort, and the potential of this tool to assist medical professionals is promising. However, our findings necessitate a cautious approach to their application in clinical and educational settings. This study serves as a call for continued research and development in this area to enhance the performance of AI tools, such as ChatGPT, in diverse linguistic and professional contexts.

## Appendix

Multimedia Appendix 1 Screenshots of the prompts in ChatGPT (GPT-4) in Korean.

Multimedia Appendix 2 Screenshots of the prompts in ChatGPT (GPT-4) in English. A) An example from the translation process. Each question in Korean was entered in succession, along with the translation command. B) The initial command of the testing process. The command was entered in English which was translated from the original Korean version. C) An example of testing ChatGPT with English questions.

Multimedia Appendix 3 The actual scores of Korean anesthesiology residents.

## Abbreviations

ABA: American Board of Anesthesiology
AI: artificial intelligence
KSA: Korean Society of Anesthesiologists

## Data Availability

The data sets generated during or analyzed during this study are available from the corresponding author upon reasonable request.
