# Supplementary material for: Performance of ChatGPT in the In-Training Examination for Anesthesiology and Pain Medicine Residents in South Korea: Observational Study
Source: JMIR Med Educ. 2024 Sep 16;10:e56859. doi: 10.2196/56859 (PMC11443200; doi:10.2196/56859)
Supplement: Multimedia Appendix 1 [file mededu_v10i1e56859_app1.pdf]

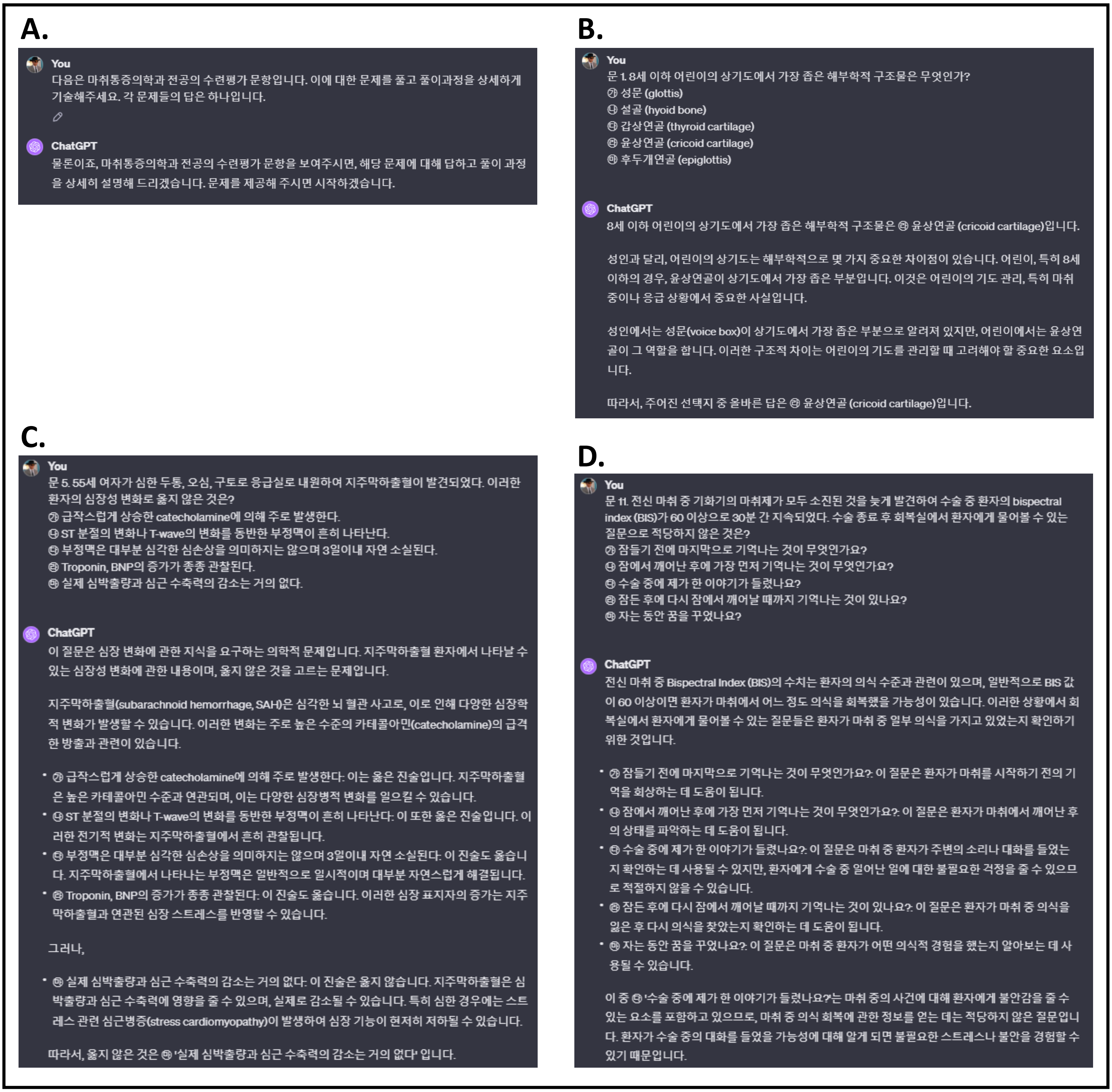

Multimedia Appendix 1. Screenshots of the prompts in ChatGPT (GPT-4) in Korean.

A) The initial command of the testing process. Following this command, the included questions were entered one by one. **B)** An example of a first-order question without a clinical case. The question required simple fact recall of the airway anatomy in children. **C)** An example of a first-order question with a clinical case. The question provided information about patient's demographics, chief complaint, and diagnosis. To select the correct answer, the question required a primary factual judgement based on medical knowledge of the provided information on diagnosis. **D)** An example of a higher-order question with a clinical case. The question presented a case of a problematic situation that could arise intraoperatively, requiring the additional thinking step to infer the implication of the situation and the methods of verifying the presumed issue.
