# Supplementary material for: Performance of ChatGPT in the In-Training Examination for Anesthesiology and Pain Medicine Residents in South Korea: Observational Study
Source: JMIR Med Educ. 2024 Sep 16;10:e56859. doi: 10.2196/56859 (PMC11443200; doi:10.2196/56859)
Supplement: Multimedia Appendix 2 [file mededu_v10i1e56859_app2.pdf]

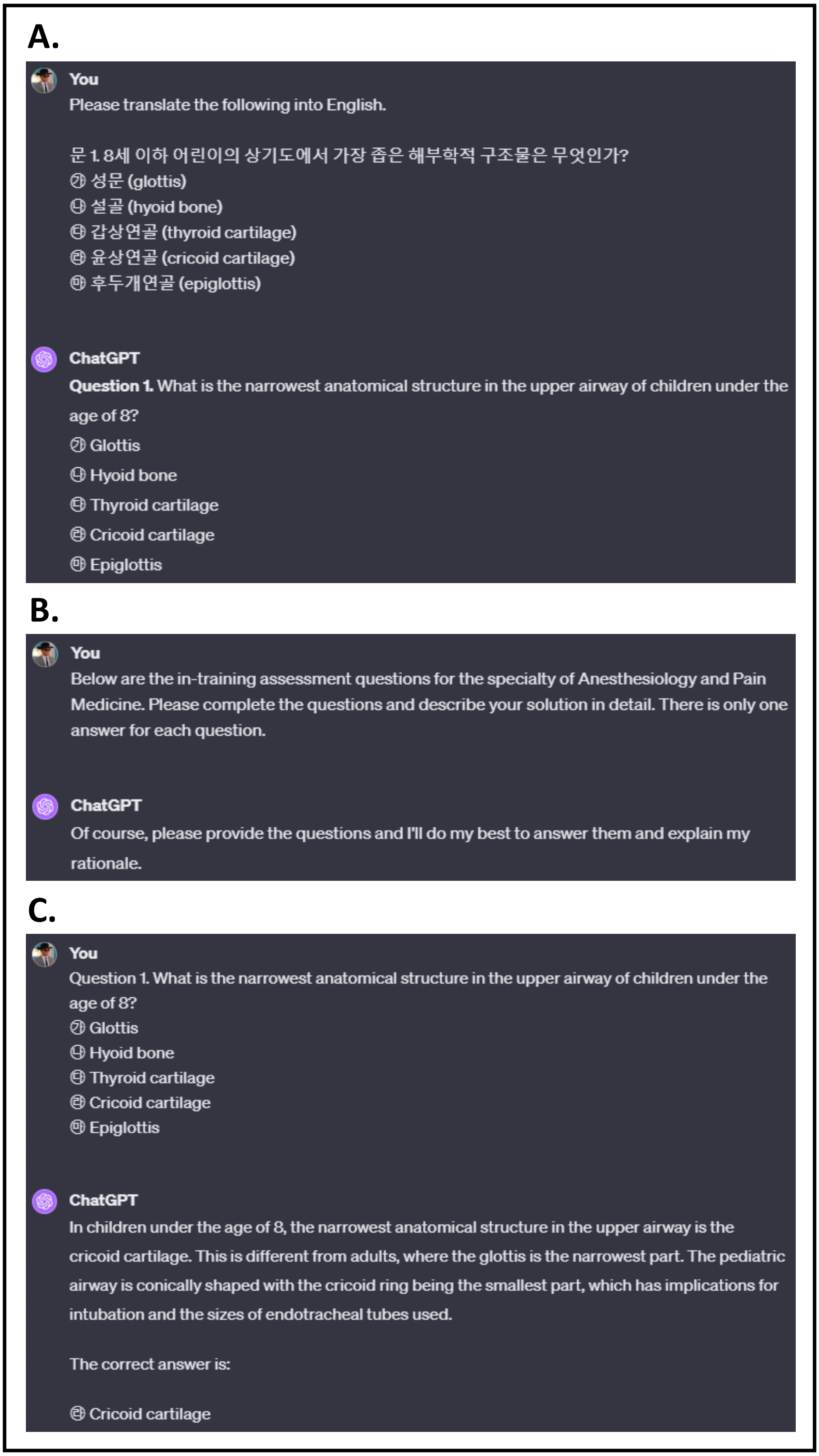

Multimedia Appendix 2. Screenshots of the prompts in ChatGPT (GPT-4) in English.

A) An example from translation process. Each question in Korean was entered one by one, along with translation command. **B)** The initial command of the testing process. The command was entered in English which was translated from the original Korean version. **C)** An example of testing ChatGPT with English question.
