# Supplementary material for: Performance of ChatGPT in the In-Training Examination for Anesthesiology and Pain Medicine Residents in South Korea: Observational Study
Source: JMIR Med Educ. 2024 Sep 16;10:e56859. doi: 10.2196/56859 (PMC11443200; doi:10.2196/56859)
Supplement: Multimedia Appendix 3 [file mededu_v10i1e56859_app3.docx]

| Year | Exam scores of residents (number of actual examinees)^*^ |
| --- | --- |
| 2022 | 62.2 ± 10.9 (1,018) |
| 2021 | 66.1 ± 11.7 (797) |
| 2019 | 60.1 ± 8.9 (802) |

**^*^**Residents’ scores are presented as the mean ± standard deviation, with the actual number of examinees for each examination in parentheses. These results were based on a full set of 100 questions annually (one point per question), and no questions were excluded.
